# Combination of Sildenafil and Ba^2+^ at a Low Concentration Show a Significant Synergistic Inhibition of Inward Rectifier Potassium Current Resulting in Action Potential Prolongation

**DOI:** 10.3389/fphar.2022.829952

**Published:** 2022-04-25

**Authors:** Martin Macháček, Olga Švecová, Markéta Bébarová

**Affiliations:** Department of Physiology, Faculty of Medicine, Masaryk University, Brno, Czechia

**Keywords:** arrhythmia, barium, cardiomyocytes, inward rectifier potassium current, sildenafil, synergy

## Abstract

Sildenafil (Viagra) is a vasodilator mainly used in the treatment of erectile dysfunction. Atrial or ventricular fibrillation may rarely occur as a side effect during sildenafil therapy. Although changes in inward rectifier potassium currents including *I*_K1_ are known to contribute to the pathogenesis of fibrillation, the effect of sildenafil on *I*_K1_ has not been studied. In experiments, Ba^2+^ is used as a specific inhibitor of *I*_K1_ at high concentrations (usually 100 µM). Being an environmental contaminant, it is also present in the human body; Ba^2+^ plasmatic concentrations up to 1.5 µM are usually reported in the general population. This study was primarily aimed to investigate changes of *I*_K1_ induced by sildenafil in a wide range of concentrations (0.1–100 µM). Additionally, the effect of combination of sildenafil and Ba^2+^ at selected clinically-relevant concentrations was tested, at 0.1 µM both on *I*_K1_ and on the action potential duration (APD). Experiments were performed by the whole-cell patch-clamp technique on enzymatically isolated rat ventricular cardiomyocytes, mostly at 23°C with the exception of APD measurements which were performed at 37°C as well. Sildenafil caused a significant, reversible, and concentration-dependent inhibition of *I*_K1_ that did not differ at −50 and −110 mV. Simultaneous application of sildenafil and Ba^2+^ at 0.1 µM revealed a massive inhibition of both inward and outward components of *I*_K1_ (this synergy was missing at other tested combinations). The combined effect at 0.1 µM (45.7 ± 5.7 and 43.0 ± 6.9% inhibition at −50 and −110 mV, respectively) was significantly higher than a simple sum of almost negligible effects of the individual substances and it led to a significant prolongation of APD at both 23 and 37°C. To our knowledge, similar potentiation of the drug-channel interaction has not been described. The observed massive inhibition of *I*_K1_ induced by a combined action of the vasodilator sildenafil and environmental contaminant Ba^2+^ at a low concentration and resulting in a significant APD prolongation may contribute to the genesis of arrhythmias observed in some patients treated with sildenafil.

## Introduction

Sildenafil (Viagra), a phosphodiesterase type 5 inhibitor, is a vasodilator used in the treatment of erectile dysfunction and pulmonary arterial hypertension (Galiè et al., 2009; Burks et al., 2018). The therapeutic plasma concentrations vary between 0.2 and 1.6 µM (Jackson et al., 1999; Croom and Curran, 2008; Gruenig et al., 2009). Although the side effects of sildenafil are usually transient and mild (Croom and Curran, 2008), serious or even fatal arrhythmias may occur. Atrial fibrillation (AF) or less often ventricular tachycardia (VT)/ventricular fibrillation (VF) were reported after sildenafil administration, mostly in patients suffering from chronic heart diseases, however, usually without arrhythmias in anamnesis (Hayashi et al., 1999; Awan et al., 2000; Rasmussen et al., 2007; Varma et al., 2012). Episodes of AF after the use of sildenafil were reported even in healthy individuals (Rasmussen et al., 2007; Ruhela and Bagarhatta, 2018). Tracqui *et al.* (2002) published a case of sudden cardiac death closely connected to a massive intake of sildenafil (post-mortem plasmatic concentration reached 13.2 µM). Fatal arrhythmia was suggested as the cause of death of the patient. Since sildenafil is mostly used by older male patients with an increased cardiovascular risk (Kessler et al., 2019), arrhythmias occurring in them may be misattributed to their cardiovascular disease. Therefore, the true prevalence of sildenafil-related arrhythmias and deaths may be higher than that reported.

Changes in inward rectifier potassium currents including *I*_K1_ are known to considerably contribute to the pathogenesis of both AF and VF (e.g., Dhamoon and Jalife, 2005; Atienza et al., 2006; Ehrlich, 2008; Heijman et al., 2014). The effect of sildenafil on *I*_K1_ has not been studied so far.

Ba^2+^ is experimentally used as a potent concentration-dependent inhibitor of *I*_K1_ (Alagem et al., 2001; Bhoelan et al., 2014). This metal is an environmental contaminant that can be identified in humans. It accumulates in the bones, teeth, heart, lungs, kidneys, and liver (Dallas and Williams, 2001; Kravchenko et al., 2014). Under standard exposure conditions, its plasmatic concentrations in the general population usually vary between 0.01 and 1.5 µM (Hung and Chung, 2004; Oskarsson and Reeves, 2007).

This study was aimed to investigate changes of the cardiac *I*_K1_ induced by sildenafil in a wide range of concentrations including the clinically-relevant concentrations. Additionally, combined action of sildenafil and Ba^2+^ at selected concentrations was analysed.

## Materials and Methods

### Ethical Approval

The experiments were carried out with respect to recommendations of the European Community Guide for the Care and Use of Laboratory Animals; the experimental protocol was approved by the Local Committee for Animal Treatment at Masaryk University, Faculty of Medicine, and by the Ministry of Education, Youth and Sports of the Czech Republic (MSMT-33846/2017-2).

### Cell Isolation

All enzymes and chemicals used during the cell isolation and measurements were purchased from Sigma-Aldrich if not stated otherwise. Cardiomyocytes were isolated from right ventricles of adult male Wistar rats (290 ± 17 g, ∼2.25 months old) anaesthetised by intramuscular administration of a mixture of tiletamine and zolazepam (65 mg/kg; Zoletil^®^ 100 inj., Virbac, France) and xylazine (20 mg/kg; Xylapan^®^ 20 mg/ml inj., Vetoquinol, Czech Republic). The dissociation procedure was previously described in detail (Bebarova et al., 2014). In short, the heart was retrogradely perfused *via* aorta with 0.9 mM CaCl_2_ Tyrode solution and then with nominally Ca^2+^-free Tyrode solution. During the first digestion step, the perfusion continued with nominally Ca^2+^-free Tyrode solution containing collagenase (type A, Roche Diagnostics GmbH, Germany, 1 mg ml^−1^) and protease (type XIV, 0.053 mg ml^−1^). In the second digestion step, protease was omitted. The enzyme solution was then washed out in two steps by perfusion with the low calcium Tyrode solutions (0.09 and 0.18 mM CaCl_2_). All solutions were oxygenated with 100% O_2_ at 37°C.

### Solutions and Chemicals

Tyrode solution with the following composition was used both during the dissociation procedure and to perfuse myocytes during the measurements (in mM): NaCl 135, KCl 5.4, MgCl_2_ 0.9, HEPES 10, NaH_2_PO_4_ 0.33, CaCl_2_ 0.9, glucose 10 (pH was adjusted to 7.4 with NaOH). To inhibit the calcium current *I*_Ca_ and the delayed rectifier potassium current *I*_K_, CoCl_2_ (2 mM) and tetraethylammonium chloride (TEA, 50 mM), respectively, were applied. Additionally, 1 µM atropine and 10 µM glybenclamide were administered to avoid a contribution of the acetylcholine-activated potassium current *I*_K(Ach)_ and the ATP sensitive potassium current *I*_K(ATP)_ to the observed *I*_K1_ changes despite it is unlikely under our experimental conditions (5 mM ATP in the pipette solution, isolated ventricular cells). Hence, the measured current should be mainly represented by *I*_K1_. During AP recordings, all specific inhibitors were omitted.

The patch electrode filling solution contained (in mM): L-aspartic acid 130, KCl 25, MgCl_2_ 1, K_2_ATP 5, EGTA 1, HEPES 5, GTP 0.1, Na_2_-phosphocreatine 3 (pH 7.25 adjusted with KOH).

CoCl_2_ and atropine were prepared as 1 M and 1 mM stock solutions, respectively, in the deionized water. Glybenclamide was prepared as 100 mM stock solution in DMSO (DMSO below 0.01% in both control and test solution). To prepare the TEA-containing stock solution, NaCl in the used Tyrode solution (described above) was replaced equimolarly by TEA.

The sildenafil stock solution (10 mM; sildenafil was dissolved in DMSO) and the Ba^2+^ stock solution (10 mM; BaCl_2_ was dissolved in deionised water) were added to the Tyrode solution to obtain sildenafil concentrations between 0.1 and 100 μM and Ba^2+^ concentrations of 0.1, 0.3, 1, and 100 μM. During simultaneous application of both drugs, the following combinations of Ba^2+^ and sildenafil were used: 0.1 μM sildenafil + 0.1 μM Ba^2+^, 1 μM sildenafil + 0.1 μM Ba^2+^, 1 μM sildenafil + 1 μM Ba^2+^, and 0.1 μM sildenafil + 0.3 μM Ba^2+^. The concentration of DMSO in the final solution was kept below 0.01% in all experiments, thus, it should not cause any changes of the cardiac *I*_K1_ by itself as mentioned above (Ogura et al., 1995; Bosch et al., 1999).

The solutions were applied into close vicinity of the measured cell through a cannula directed on the cell and distant from it by about 100–150 µm *via* a gravity-operated perfusion system; the time to change the solution in the surroundings of the measured cells was approximately 2 s.

### Electrophysiological Measurements and Evaluation

Single rod-shaped cells with well visible striations were used for the membrane current and voltage recordings applying the whole-cell patch-clamp technique in the voltage-clamp and current-clamp modes, respectively. The patch pipettes were pulled from borosilicate glass capillary tubes and heat polished on a programmable horizontal puller (Zeitz-Instrumente, Germany). The resistance of the filled glass electrodes was below 1.5 MΩ to keep the access resistance as low as possible. For the generation of experimental protocols and data acquisition, the Axopatch 200B amplifier, Digidata 1322A, and pCLAMP 9.2 software (Molecular Devices, United States) were used. The series resistance was compensated up to 75%. The measured ionic currents and APs were digitally sampled at 10 kHz and stored on the hard disc. Experiments were performed mostly at room temperature (23 ± 1°C), just some AP recordings were performed at 37°C. The holding potential was −85 mV, and the stimulation frequency was 0.2 Hz in all experiments on *I*_K1_. During AP recordings, the measured cell was stimulated at 1 Hz. *I*_K1_ was evaluated as the current sensitive to 100 µM Ba^2+^ at the end of 500-ms pulse, both to −50 mV (the sodium current *I*_Na_ was inactivated at the beginning of this pulse) and to −110 mV to check the outward and inward current component, respectively. Additional recordings during a 3-s voltage ramp from −110 to −10 mV were performed as well.

### Statistical Analysis

The results are presented as arithmetic means ± S.E.M. (normality of the data distribution tested by the Kolmogorov-Smirnov test, K-S test) from *n* cells (Origin, version 8.5.1, Origin Lab Corporation). The curve fitting paired and unpaired *t*-test, and ANOVA test (with the Tuckey´s post-test) were performed using the GraphPad Prism, version 6.05 (GraphPad Software, Inc.); *p* < 0.05 was considered statistically significant.

## Results

**FIGURE 1:**
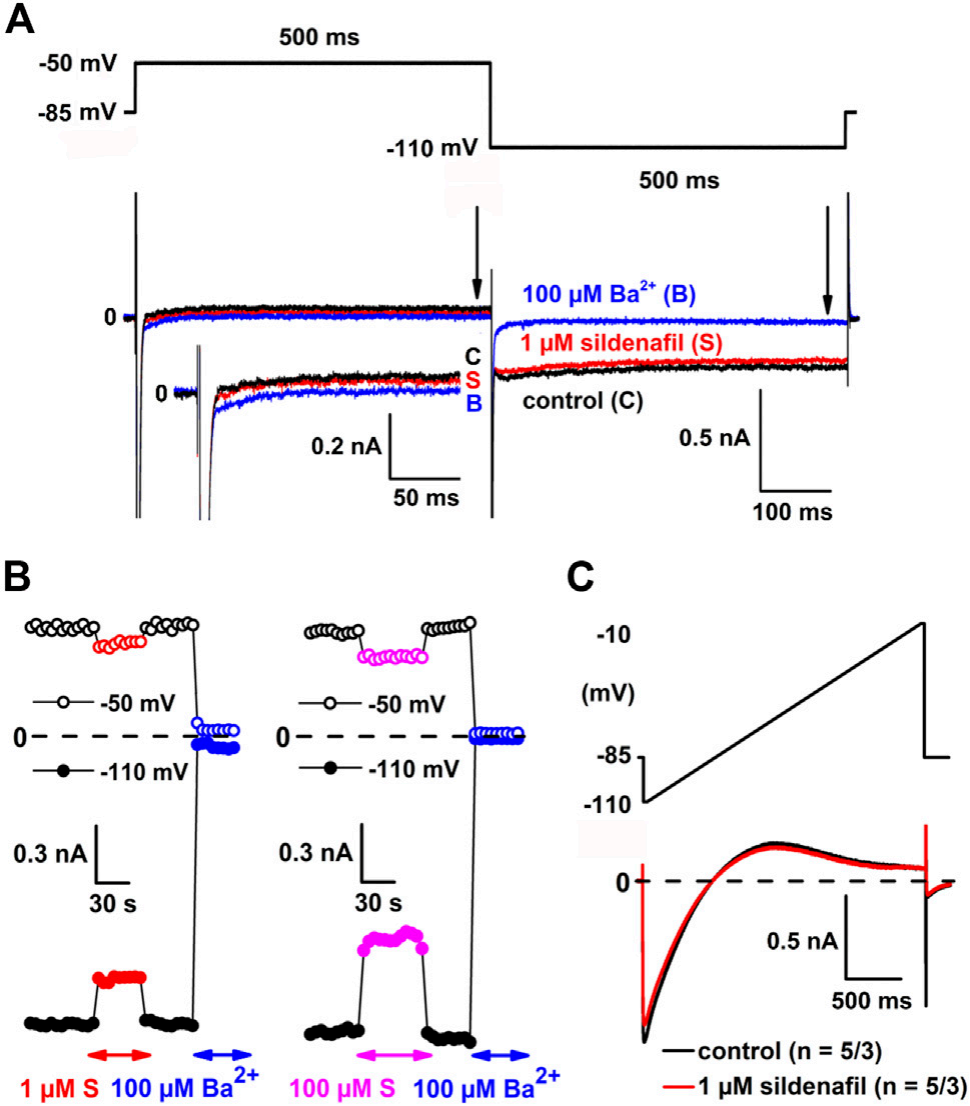
Sildenafil inhibits inward rectifier potassium current (*I*_K1_). **(A)** The experimental protocol (upper panel) and representative current traces in control conditions (C), under the effect of 1 µM sildenafil (S) and 100 µM Ba^2+^ (B) at −50 mV and −110 mV (lower panel); inset: current traces at −50 mV in detail; arrows indicate the approximate time of analysis of *I*_K1_ magnitude. **(B)** Changes of *I*_K1_ at −50 mV and −110 mV during subsequent applications of 1 µM sildenafil and 100 µM Ba^2+^ (left panel) and the highest sildenafil concentration 100 µM (right panel). **(C)** Average current-voltage relationship in control and under the effect of 1 µM sildenafil (*n* = 5/3).

Sildenafil at a representative therapeutic concentration of 1 µM partially inhibited *I*_K1_ at both −50 and −110 mV (Figure 1A; for the used experimental protocol, see the upper panel). The inhibition proceeded fast (the steady-state effect was reached within 7.5 ± 2.5 s on average; *n* = 15/8 and 16/8 at −50 and −110 mV, respectively) and was fully reversible during the subsequent wash-out (Figure 1B, left panel). A similar time course of the inhibition and wash-out was observed in other tested concentrations (for an example at the highest tested sildenafil concentration, see Figure 1B, right panel). Figure 1C shows the average current-voltage relationship in control and under the effect of 1 µM sildenafil (*n* = 5/3). The magnitude of control *I*_K1_ and the cell membrane capacitance significantly correlated (not illustrated; the Pearson correlation coefficient was 0.50 and −0.39 at −50 and −110 mV, respectively, *p* < 0.05). The evaluated current is therefore further expressed as the current density (in pA/pF) to reduce differences among cells caused by their varying size.

**FIGURE 2:**
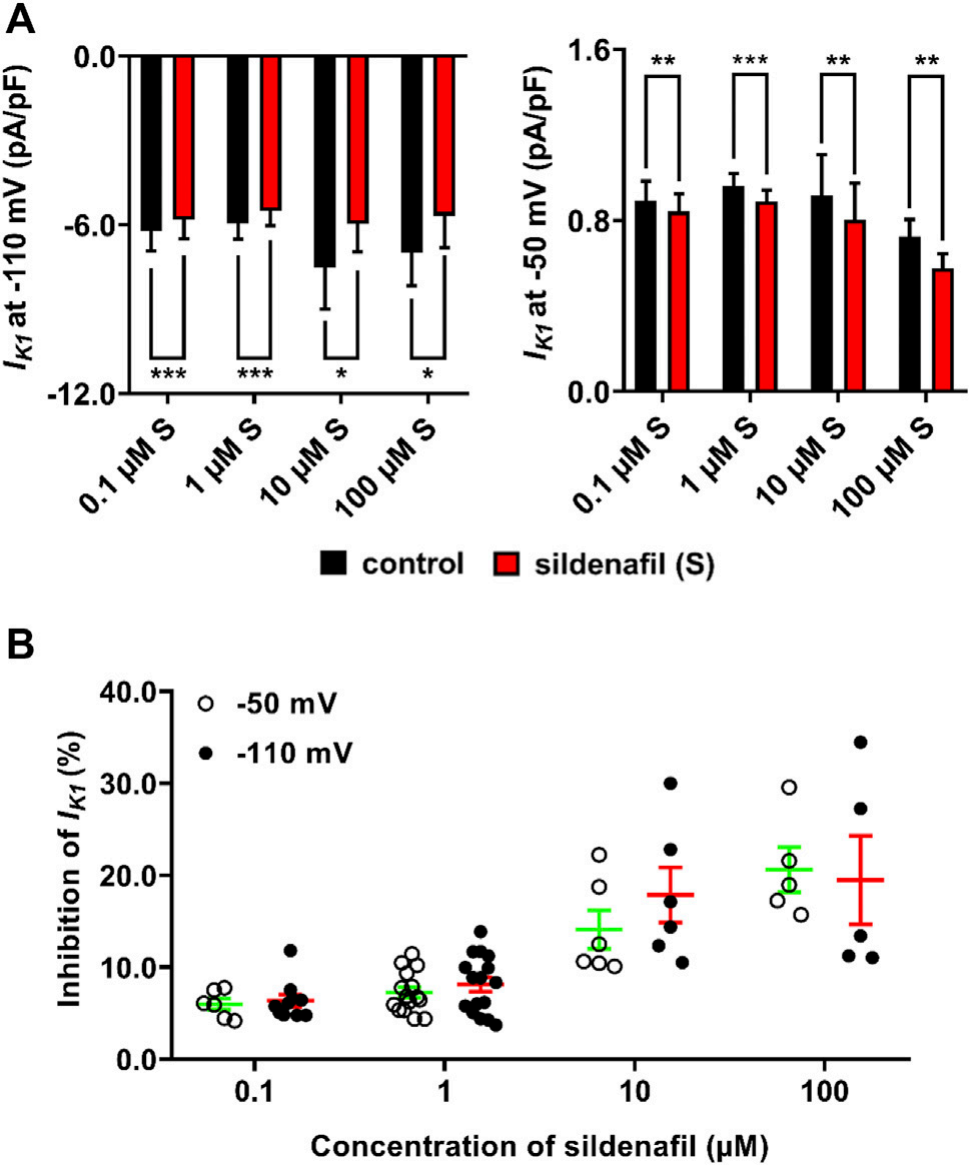
Average effects of sildenafil on *I*_K1_. **(A)** Average *I*_K1_ density in control and under the effect of sildenafil between 0.1 and 100 μM at −110 mV (left graph) and −50 mV (right graph); paired *t*-test, *, ** and ***—statistically significant difference at *p* < 0.05, 0.01 and 0.001, respectively, (no significant differences among control values at the respective voltage). **(B)** Concentration dependence of the inhibition by sildenafil was present at both −50 mV and −110 mV; no significant difference between effects at both tested voltages was observed.

The average effects of sildenafil at concentrations between 0.1 and 100 μM on *I*_K1_ density are shown in Figure 2 *.* Sildenafil elicited a statistically significant decrease of *I*_K1_ in all used concentrations (*p* < 0.05). The subclinical 0.1 μM sildenafil evoked a mild inhibition of *I*_K1_ by 6.0 ± 0.6% (*n* = 6/6) and 6.4 ± 0.7% (*n* = 10/8) at −50 and −110 mV, respectively. The therapeutic 1 μM sildenafil inhibited *I*_K1_ by 7.3 ± 0.6 (*n* = 15/8) and 8.1 ± 0.8 (*n* = 16/8) at −50 and −110 mV, respectively. The supratherapeutic concentrations of sildenafil gave rise to a more potent decrease of *I*_K1_ (14.1 ± 2.1 and 17.9 ± 3.0% inhibition by 10 μM sildenafil, *n* = 6/6, and 20.6 ± 2.4 and 19.5 ± 4.8% inhibition by 100 μM sildenafil, *n* = 5/5, at −50 and −110 mV, respectively, Figure 2A). The reduction of *I*_K1_ was significantly more profound when the higher sildenafil concentrations were applied, both at −50 mV and −110 mV (Figure 2B; *p* < 0.05). No statistically significant difference was found between the sildenafil effects at −50 and −110 mV.

**FIGURE 3:**
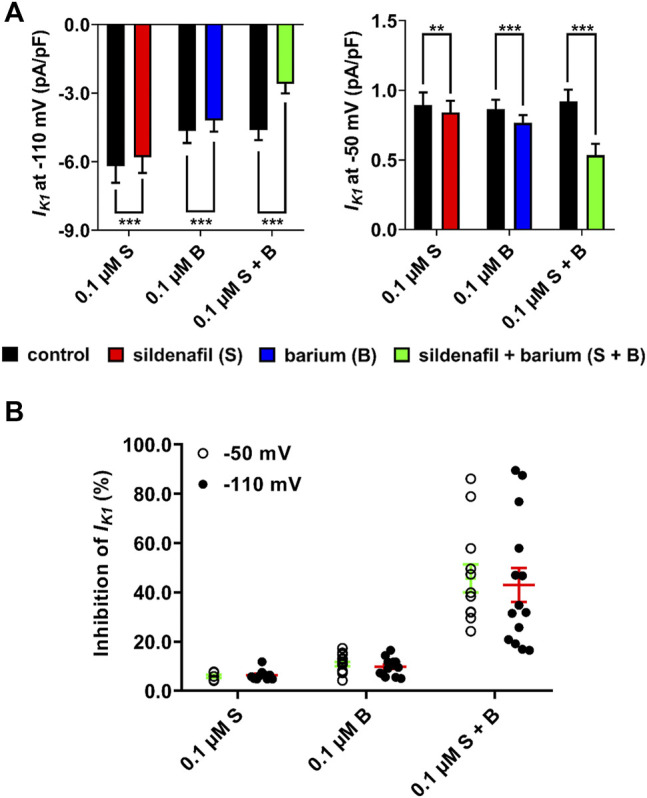
Synergistic inhibitory effect of sildenafil and Ba^2+^ at a low concentration of 0.1 µM. **(A)** Changes of *I*_K1_ in the presence of sildenafil and Ba^2+^, applied both separately and in combination; paired *t*-test, *, ** and ***—statistically significant difference at *p* < 0.05, 0.01 and 0.001, respectively. **(B)** Massive inhibition of *I*_K1_ appeared after combined application of both sildenafil and Ba^2+^. The inhibition was significantly more potent than a simple sum of separate effects of sildenafil and Ba^2+^. The effect was not significantly different between voltages.

Subsequently, changes of *I*_K1_ after both separate and combined application of 0.1 μM sildenafil and 0.1 μM Ba^2+^ were analysed (Figure 3)*.* As described above, the inhibition of *I*_K1_ by the subclinical 0.1 μM sildenafil was only mild, but significant at both voltages. The inhibitory effect of Ba^2+^ at a clinically-relevant concentration of 0.1 μM was statistically significant (*p* < 0.05) both at −50 mV (10.9 ± 0.9, *n* = 16/5) and at −110 mV (9.8 ± 0.9, *n* = 15/6). Combined application of sildenafil and Ba^2+^ significantly and massively reduced *I*_K1_ by 45.7 ± 5.7 (*n* = 12/3) and 43.0 ± 6.9% (*n* = 14/5) at −50 and −110 mV, respectively, (Figure 3A; *p* < 0.05). The effect of the combined solution was significantly more potent than a simple sum of effects of separate solutions (Figure 3B; *p* < 0.05). No statistically significant difference between the effects at the tested voltages was observed. The high variability of the combined action of sildenafil and Ba^2+^ (Figure 3B) is likely related to the varying composition and, thus, properties of *I*_K1_ channel heterotetramers in individual measured cells (see Discussion).

**FIGURE 4:**
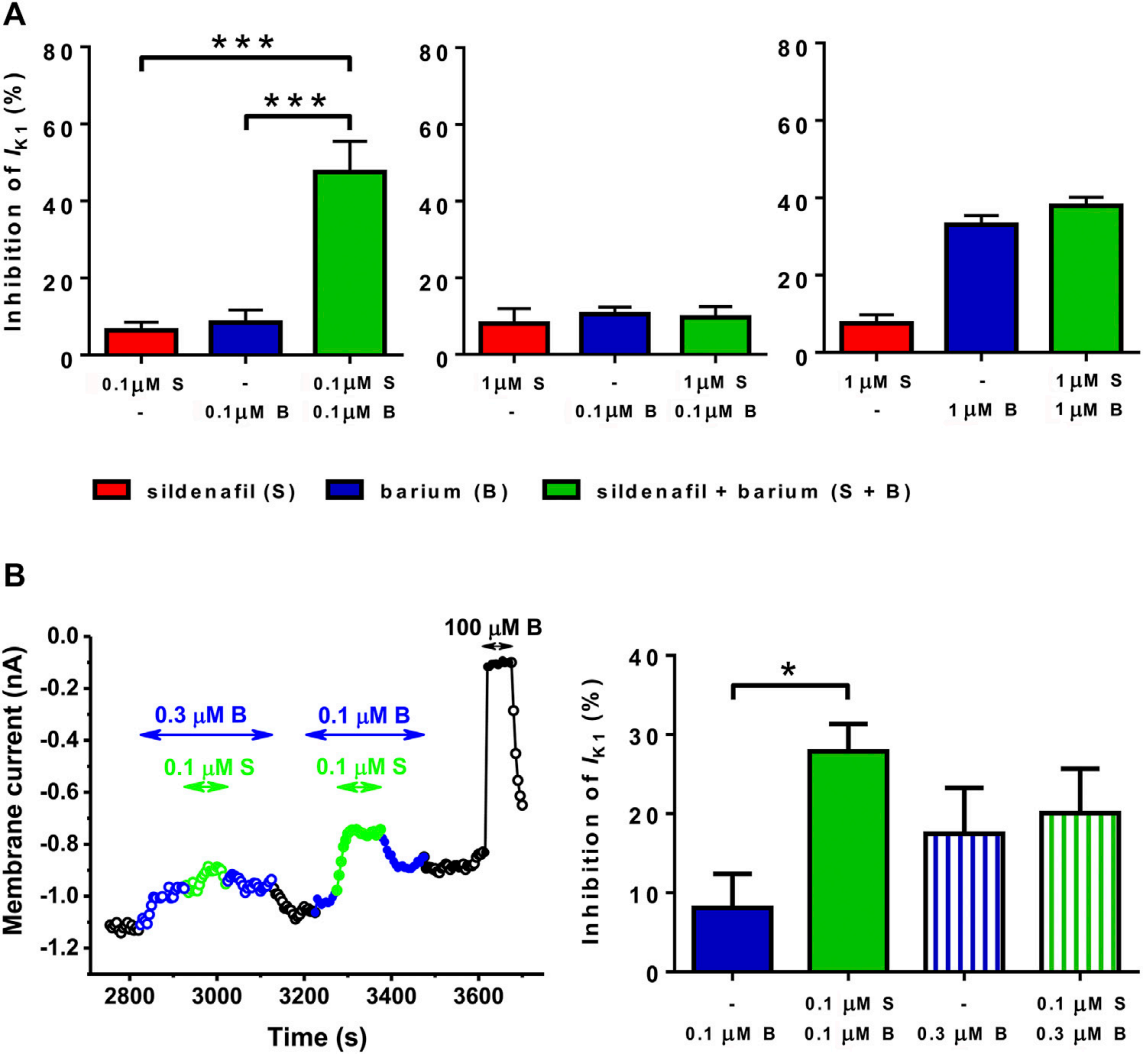
Combined action of sildenafil (S) and Ba^2+^ (B) at selected concentrations. **(A)** The synergy between sildenafil and Ba^2+^ was observed only in the case of combination of 0.1 µM sildenafil + 0.1 µM Ba^2+^ (*n* = 7–11/4−8); ***—statistical significance at *p* < 0.001. **(B)** Paired comparison of the effect of 0.1 µM Ba^2+^, 0.1 µM sildenafil + 0.1 µM Ba^2+^, 0.3 µM Ba^2+^, and 0.1 µM sildenafil + 0.3 µM Ba^2+^ (left panel–representative recording; right panel–average data, *n* = 4/1); *—statistical significance at *p* < 0.05.

Several combinations of sildenafil and Ba^2+^ at various concentrations were then tested to elucidate concentration dependence of the synergistic effect described above. As apparent, the combination of 0.1 μM sildenafil + 0.1 μM Ba^2+^ seems to be unique because it was the only one causing the synergistic effect (Figure 4A, left panel). If 0.1 μM Ba^2+^ was combined with 1 μM sildenafil (Figure 4A, middle panel) or if 1 μM Ba^2+^ was combined with 1 μM sildenafil (Figure 4A, right panel), no significant increase of the effect was present in comparison with the effect of 0.1 and 1 μM Ba^2+^ and 1 μM sildenafil alone, respectively. We also investigated a paired comparison of two combinations of the substances, the originally tested combination of 0.1 μM sildenafil + 0.1 μM Ba^2+^ and a new combination of 0.1 μM sildenafil + 0.3 μM Ba^2+^. Figure 4B demonstrates that no synergistic effect under the combined action of 0.1 μM sildenafil + 0.3 μM Ba^2+^ could be revealed, despite the originally tested combination of 0.1 μM sildenafil + 0.1 μM Ba^2+^ induced a clear synergistic effect in the same cell (*n* = 4/1, *p* < 0.05).

**FIGURE 5:**
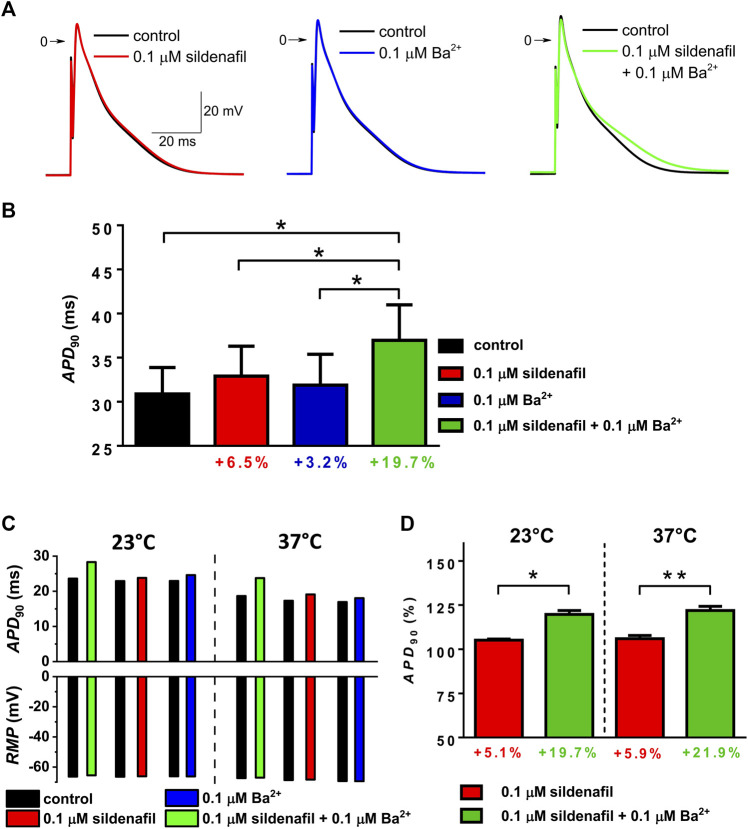
Changes of action potential duration (APD) under the effect of 0.1 µM sildenafil alone and in combination with Ba^2+^ (0.1 µM); APD was evaluated at 90% repolarization (*APD*_90_). **(A)** Representative AP waveforms in control and under the effect of the drugs alone and in combination at 23°C. **(B)** Average *APD*_90_ changes at 23°C (*n* = 7/3). **(C)** Values of *APD*_90_ and resting membrane potential (*RMP*) in a representative cell measured both at 23 and 37°C. **(D)** Comparison of the average relative *APD*_90_ changes under the effect of sildenafil in the absence and presence of Ba^2+^ at 23 and 37°C (*n* = 4/2); * and **—statistical significance at *p* < 0.05 and 0.01, respectively.

To find out the impact of the observed *I*_K1_ changes on cardiac cell repolarization, action potentials (APs) were recorded at separate and combined application of sildenafil and Ba^2+^, both at 0.1 μM. In contrast to *I*_K1_ recordings, these measurements were performed in the absence of any specific inhibitors. As clearly demonstrated using the representative AP recordings in Figure 5A, the action potential duration (APD) was markedly prolonged in presence of both sildenafil and Ba^2+^, but not if each of the drugs was applied alone; changes of other parameters of APs were not apparent. APD evaluated at 90% repolarization (*APD*_90_; Figure 5B, *n* = 7/3) was not significantly changed if sildenafil and Ba^2+^ were applied separately, but it was significantly higher in their combined presence (*p* < 0.05). The combined effect was significantly higher than the effect of sildenafil and Ba^2+^ alone (Figure 5B; *p* < 0.05) which is in a good agreement with *I*_K1_ data presented in Figure 3. The synergistic effect of sildenafil and Ba^2+^ on APD was also preserved, even up to a comparable extent, at physiological temperature (Figures 5C, D, *n* = 4/2).

## Discussion

This is the first study reporting the effect of sildenafil on the cardiac Kir channels. Sildenafil caused a significant and reversible concentration-dependent inhibition of *I*_K1_ at −110 mV and −50 mV, even at clinically-relevant concentrations. Surprisingly, simultaneous application of subclinical concentration of sildenafil (0.1 μM) and low clinically-relevant concentration of Ba^2+^ (0.1 μM) massively decreased both inward and outward components of *I*_K1_ and resulted in a significant action potential prolongation, even at physiological temperature. The combined effect was significantly higher than a simple sum of effects of the individual substances at both tested voltages. To our best knowledge, similar synergistic effect of a drug and Ba^2+^ (or other ions) has not been described.

*I*_K1_ channels are homo- and heterotetramers formed by individual Kir2x subunits, namely Kir2.1, Kir2.2, and Kir2.3 in mammalian hearts. The channels formed by various combinations of these subunits may exert different properties including different sensitivity to drugs. Even the inhibitory effect of Ba^2+^ differs in the individual Kir2x subunits and their various combinations (Schram et al., 2003). The expression profile for Kir2x isoforms in the human cardiac right ventricle is Kir2.1 > Kir2.2 > Kir2.3 (Reilly and Eckhardt, 2021). Similar pattern can be seen in rodent ventricles (Panama et al., 2007). In general, Kir2x expression patterns display very little variance among species investigated thus far (De Boer et al., 2010). Hence, we expect that changes of *I*_K1_ observed in this study on isolated rat cardiomyocytes should be applicable in human. We plan to verify this in our future study.

Changes of *I*_K1_ are known to affect the action potential duration (APD). As well known, *I*_K1_ inhibition may prolong APD, which may lead to the occurrence of early afterdepolarizations (EADs) in cardiomyocytes. In atria, EADs may trigger ectopic beats in pulmonary veins or other foci, which may initiate AF (Tse, 2016). In ventricles, prolonged APD may result in the long QT syndrome with a high risk of fatal arrhythmias such as polymorphic VT, including *torsades de pointes*. Hence, the combined action of sildenafil and Ba^2+^ observed in this study, inhibiting almost half of *I*_K1_ (Figure 3), may contribute to arrhythmogenesis.

No changes of APD in isolated cardiomyocytes have been observed at therapeutic sildenafil concentrations in previous animal studies (Geelen et al., 2000; Chiang et al., 2002). Beside the potential role of Ba^2+^ (which may be absent in laboratory animals), it might reflect effect of sildenafil on more cardiac ionic channels which can mutually compensate for their effect on APD. Chiang *et al.* (2002) discovered that sildenafil dose-dependently inhibited the depolarizing L-type calcium current *I*_Ca_ in guinea-pig ventricular myocytes, at least slightly even at the therapeutic concentration of 1 μM (which might compensate the inhibitory effect of sildenafil on *I*_K1_ observed in our study). A significant shortening of APD (presumably caused by *I*_Ca_ inhibition) was observed in supratherapeutic sildenafil concentrations above 10 μM (Chiang et al., 2002). Sildenafil was also shown to inhibit delayed rectifier potassium current (*I*_Kr_; the human hERG channels expressed in a cell line), but only at supratherapeutic concentrations (a half inhibition at 33.3 μM, Dustan Sarazan et al., 2004). In agreement, Geelen *et al.* (2000) pointed out that sildenafil significantly inhibited the human hERG channels expressed in a cell line and prolonged APD in guinea-pig isolated hearts at supratherapeutic concentrations above 30 μM. Anyway, our experiments proved a significant AP prolongation during combined application of 0.1 µM sildenafil and 0.1 µM Ba^2+^ by about 20% both at the room temperature of 23°C and at the physiological temperature of 37°C (Figure 5). Hence, proarrhythmic changes under the effect of sildenafil should be considered. It might explain occurrence of arrhythmias in some patients treated with sildenafil, namely those with an accumulation of Ba^2+^.

The therapeutic plasma concentration of sildenafil (1 μM) elicited only a mild inhibitory effect on *I*_K1_ (Figures 1, 2). However, little is known about precise sildenafil concentration in the human heart *in vivo*. Sildenafil is a lipophilic agent with high distribution volume (105 L; Chaumais et al., 2013; Nichols et al., 2002; Langtry and Markham, 1999), that much exceeds average total body water volume (42 L). This attribute implies considerable distribution to body tissues (Nichols et al., 2002). Elimination of sildenafil takes place in the liver *via* enzymes CYP3A4 and CYP2C9. When certain conditions are met (higher drug dosage, liver failure, administration of CYP inhibitors), the peak plasmatic concentration of sildenafil may increase up to 3.9-fold and the drug cumulates in body tissues (Muirhead et al., 2000; Hyland et al., 2001). Since the effect of sildenafil is concentration-dependent, such an increase in the drug levels might lead to a more potent inhibition of *I*_K1_ (and indeed also *I*_Ca_ and *I*_Kr_), thus, significant changes of APD with subsequent arrhythmogenesis might appear.

A synergistic inhibitory effect of a subclinical concentration of sildenafil and a low clinically-relevant concentration of Ba^2+^ on *I*_K1_ was revealed (Figure 3). The underlying mechanism remains unclear, although Ba^2+^ alone is a specific dose-dependent inhibitor of *I*_K1_ (Bhoelan et al., 2014). Higher exposition to this metal is possible in industrial areas where it may enter human bodies *via* contaminated air, water, and food. Ba^2+^cumulate mainly in bones, but slightly also in internal organs including the heart (Kravchenko et al., 2014). As well known, the plasma Ba^2+^ concentrations above 2.5 μM considerably increase the risk of arrhythmias including VF (Kravchenko et al., 2014; Bhoelan et al., 2014; Oskarsson and Reeves, 2007). Since Ba^2+^ alone in higher concentrations is proarrhythmic and since the significant synergistic inhibitory effect of 0.1 µM sildenafil and 0.1 µM Ba^2+^ on *I*_K1_ was observed, we suggest that people in areas contaminated by Ba^2+^ may be more susceptible to arrhythmias after administration of sildenafil than the common population. Unfortunately, there is no direct evidence to support the claim since the current area distribution of sildenafil-related arrhythmias is not known. Moreover, the synergistic effect seems to be uniquely present just in a very specific combination(s) of sildenafil and Ba^2+^ (Figure 4), thus, occurrence of arrhythmias based on this synergistic effect is likely rare. Similar synergy between the effect of Ba^2+^ and other two inhibitors of phosphodiesterase type 5 used in clinical practice, vardenafil and tadalafil, cannot be excluded. Tadalafil seems to have negligible effects on QTc interval in human, being comparable to placebo (Beasley et al., 2005). In contrast, vardenafil slightly prolonged QTc interval at both therapeutic and supratherapeutic dose in human as documented by Morganroth *et al.* (2004). To our knowledge, the mechanism is not known (hERG inhibition is not a likely cause, Dustan Sarazan et al., 2004). Regarding arrhythmias during vardenafil treatment, we have found only a case report of atrial fibrillation that was likely attributed to a reflex tachycardia induced by hypotension as concluded by the authors (Veloso and de Paola, 2005). The possible synergy between these drugs and Ba^2+^ should be tested in the future.

Regarding the mechanism of synergistic action of sildenafil and Ba^2+^ on *I*_K1_ and its unique presence only at a low concentration of the substances (Figures 3, 4), we can just speculate. Considering the size and character of the sildenafil molecule which is relatively big and lipophilic, we expect its binding either on the outer channel pore or on the cytoplasmatic portion of the channel. This might cause an allosteric conformational change (modulation) of the channel structure, thus, tighter binding of Ba^2+^ within the channel pore. Two main residues were shown to affect the inhibitory effect of Ba^2+^ on *I*_K1_, E125 located at the outer channel vestibule and T141 located close to the selectivity filter (Alagem et al., 2001). These lie close to the presumed aforementioned allosteric modulation sites. Other Kir2.1 channel residues may also affect the sensitivity of the channel to Ba^2+^-induced inhibition, for example the residue at the position 121 (Zhou et al., 1996). Absence of the synergy at higher concentrations might be theoretically related to a competitive inhibition of the channel by sildenafil and Ba^2+^ which would disable binding of another molecule if the site was already occupied by the other one. To reveal the specific binding site of sildenafil on *I*_K1_ channel and explain mechanism of the synergistic effect of sildenafil and Ba^2+^, a separate study is planned in the future, encompassing several techniques, namely the structural modelling, site directed mutagenesis, and patch-clamp technique.

We conclude that sildenafil caused a significant, reversible, and concentration-dependent inhibition of both inward and outward components of *I*_K1_ even at therapeutic concentrations. The observed massive inhibition of *I*_K1_ induced by simultaneous application of sildenafil and Ba^2+^ at a low concentration and resulting in a significant AP prolongation is unique, not described in the literature so far according to our knowledge. Since both sildenafil and Ba^2+^ can accumulate in the human body tissues, we presume that, when certain conditions are met, the use of sildenafil and the related changes in *I*_K1_ might result in arrhythmia.

## Data Availability

The raw data supporting the conclusions of this article will be made available by the authors, without undue reservation.
